# Benchmarking variational AutoEncoders on cancer transcriptomics data

**DOI:** 10.1371/journal.pone.0292126

**Published:** 2023-10-05

**Authors:** Mostafa Eltager, Tamim Abdelaal, Mohammed Charrout, Ahmed Mahfouz, Marcel J. T. Reinders, Stavros Makrodimitris

**Affiliations:** 1 Delft Bioinformatics Lab, Delft University of Technology, Delft, The Netherlands; 2 Department of Radiology, Leiden University Medical Center, Leiden, The Netherlands; 3 Department of Human Genetics, Leiden University Medical Center, Leiden, The Netherlands; 4 Leiden Computational Biology Center, Leiden University Medical Center, Leiden, The Netherlands; 5 Department of Medical Oncology, Erasmus Medical Center, Rotterdam, The Netherlands; Chinese Academy of Sciences, CHINA

## Abstract

Deep generative models, such as variational autoencoders (VAE), have gained increasing attention in computational biology due to their ability to capture complex data manifolds which subsequently can be used to achieve better performance in downstream tasks, such as cancer type prediction or subtyping of cancer. However, these models are difficult to train due to the large number of hyperparameters that need to be tuned. To get a better understanding of the importance of the different hyperparameters, we examined six different VAE models when trained on TCGA transcriptomics data and evaluated on the downstream tasks of cluster agreement with cancer subtypes and survival analysis. We studied the effect of the latent space dimensionality, learning rate, optimizer, initialization and activation function on the quality of subsequent downstream tasks on the TCGA samples. We found *β*-TCVAE and DIP-VAE to have a good performance, on average, despite being more sensitive to hyperparameters selection. Based on these experiments, we derived recommendations for selecting the different hyperparameters settings. To ensure generalization, we tested all hyperparameter configurations on the GTEx dataset. We found a significant correlation (*ρ* = 0.7) between the hyperparameter effects on clustering performance in the TCGA and GTEx datasets. This highlights the robustness and generalizability of our recommendations. In addition, we examined whether the learned latent spaces capture biologically relevant information. Hereto, we measured the correlation and mutual information of the different representations with various data characteristics such as gender, age, days to metastasis, immune infiltration, and mutation signatures. We found that for all models the latent factors, in general, do not uniquely correlate with one of the data characteristics nor capture separable information in the latent factors even for models specifically designed for disentanglement.

## Introduction

Advancements in sequencing technologies have enabled profiling different “-omics” that revolutionised the understanding of biology. These omics are usually of high dimensionality, which complicates the data analysis. This has sparked a large research interest in dimensionality reduction methods which represent data in a lower-dimensional space while reducing noise and preserving the signal in the data. There are different dimensionality reduction methods that can be categorized into linear and non-linear methods [1, 2]. Selecting an appropriate dimensionality reduction method for an application depends on the structure of the high-dimensional space and the structure of the low-dimensional manifold that we assume that the data belongs to.

**Fig 1:**
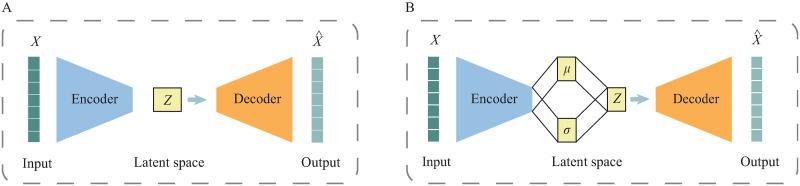
Schematics for autoencoder and variational autoencoder. Both models are based on the encoder-decoder neural network structure to a learn latent space. A) An autoencoder is a deterministic model where *z* is a mapping of the input data. B) A variational autoencoder is a probabilistic model where the mapping *z* is generated by a probability distribution conditioned on the input data.

Variational AutoEncoders (VAE) are among the most used methods nowadays to embed omics data into a lower dimensional representation. A variational autoencoder is similar to an autoencoder (AE) as they both learn a set of latent variables *z* to encode an input sample *x* and by forcing *z* to be able to reconstruct *x* (i.e. $\hat{x}$). Both VAE and AE are based on an encoder-decoder structure of artificial neural networks (Fig 1A and 1B). An AE is a deterministic model that is trained by minimizing the reconstruction error of the input data.

The VAE differs in that, it learns a probabilistic mapping from *x* to *z* (i.e. a probability distribution *p*(*z*|*x*)) which enables the generation of new data points by drawing samples from this distribution. Calculating this probability distribution *p*(*z*|*x*) is intractable, especially in high dimensional data. To overcome this, Kingma and Welling applied variational inference and neural networks to estimate it by a tractable approximation *q*(*z*|*x*) (see Eq 1) [3].

Various VAE variants have been proposed to address different aspects of the VAE formulation and to improve the training of VAEs on specific tasks [4]. One task gaining attention is the interpretability of the learned latent space. Several models have attempted to generate an interpretable latent space by forcing individual latent factors to correspond to specific factors of variation within the dataset, such as biological processes or metadata. Such representations are called disentangled representations [5, 6]. This definition can be generalized to a “set of latent factors” that together encode one independent factor of variation [7]. Another definition mandates the disentanglement representation to be informative, separable from each other, and interpretable [8]. Different VAE variants have been designed to tackle the disentanglement problem and claim achieving a better performance in learning a more disentangled latent space [9–11]. Some studies show that the more interpretable the latent space, the better the model is at representing the data [5, 6, 12]. For instance, Way and Greene showed that a VAE can learn a meaningful latent space trained on RNA-Seq data from The Cancer Genome Atlas (TCGA) [13, 14]. Also, VAEs are proven to be useful in several applications, such as predicting drug response [15] and perturbation effects [16]. Using a semi-supervised approach and a VAE, Wei and Ramsey were able to predict response to chemotherapy for some cancer types [17].

Despite these promising results, it is known that VAEs suffer from sensitivity to the hyperparameters, such as the learning rate, the number of hidden layers, the optimizer and the number of neurons in each layer [18–20]. Although VAEs are getting more and more widely-used, there is a lack of guidelines for selecting training hyperparameters. In addition, there has been no consistent comparison of different VAE models on their ability to learn a disentangled latent space when applied to embed RNA sequencing data of cancer patients [21].

In this paper we study the capability of different VAE models to learn the latent representation of the data and their ability to disentangle this representation. We benchmark the performance of six different VAE models and five different hyperparameters: dimensionality of the latent space, learning rate, optimizer, initialization, and activation function, leading to a total of 6,480 different VAE configurations. The performance was evaluated on the clustering quality of transcriptomic samples and the prediction of overall survival in the TCGA dataset which comprises of patients with different cancer types. To assess the generalizability of the choice of the hyperparameters, we evaluated all hyperparameter configurations on the Genotype-Tissue Expression (GTEx) dataset. Moreover, for well-performing hyperparameter configurations, we tested the disentanglement of the learned latent space. Finally, based on our benchmarks, we provide recommendations on selecting VAE models and their hyperparameters when dealing with transcriptomic data. Among the numerous VAE variants that have been proposed, we decided to focus on six models, placing our emphasis on latent space disentanglement. Vanilla VAE served as our baseline and we included other models that improve upon it on various aspects: *β*-VAE [9], *β*−TCVAE [10] and DIP-VAE [11] modified the VAE loss function in different ways to force learning of disentangled representations. The categorical VAE takes a different approach and learns a discrete latent space, where samples can be easily classified to distinct categories [22]. Finally, IWAE does not aim at interpretability, but rather at learning a richer representation, which is achieved by maximizing a tighter bound of the marginal data log-likelihood [23]. By covering these prominent directions in VAE research, our study comprehensively examines various strategies aimed at advancing the capabilities of VAEs. To help in replicating the results or testing new models or metrics, we made the code used in this study available on https://github.com/meltager/vae_benchmark.

## Materials and methods

### VAE models

#### Vanilla VAE

The VAE model aims to find a probabilistic distribution *p*(*z*|*x*) which maps the input *x* to a set of latent variables *z*. Because *p*(*z*|*x*) is intractable in most cases, we follow Kingma and Willing [3] and approximate it by a distribution *q*(*z*|*x*) with parameters *ϕ* which is approximated by a neural network (encoder). A decoder neural network then tries to reconstruct the input data from the latent variables by learning the distribution *p*(*x*|*z*) with parameters *θ* [3]. VAE achieves this by maximizing the evidence lower bound (ELBO) which is a lower bound of the data log-likelihood (*p*(*x*)) [24]. This leads to the following loss function which is the negative of the ELBO [3]:

$$\begin{array}{c} L\left(\theta ,\phi ;x\right)=-E_{q(z|x)}\left[log\;p\left(x|z\right)\right]+D_{KL}\left(q\left(z|x\right)\;||\;p\left(z\right)\right) \end{array}$$ (1)

Then, a stochastic gradient variational Bayes estimator [3] is used to minimize this loss with respect to the parameters. The first term in Eq 1 corresponds to the reconstruction error which directs the decoder to learn how to accurately reconstruct the input data. The second term is the Kullback-Leibler (KL) divergence between the learned embedding distribution of an input sample and the prior distribution *p*(*z*) which acts as a regularizer for the encoder.

#### *β*-VAE

Higgins et al. introduced the idea of learning a disentangled representation using an adaptation of a VAE called *β*-VAE. This work showed that adjusting the balance between the reconstruction loss and the KL divergence terms can push the encoder to learn a disentangled representation [9]. Thus, this model multiplies the KL term with a hyperparameter *β*, such that the loss function becomes:

$$\begin{array}{c} L\left(\theta ,\phi ;x\right)=-E_{q(z|x)}\left[log\;p\left(x|z\right)\right]+\beta \;D_{KL}\left(q\left(z|x\right)\;||\;p\left(z\right)\right) \end{array}$$ (2)

The loss function is similar to Eq 1 except for the *β* parameter. When setting *β* > 1, the model is “encouraged” to learn a disentangled representation of the training data [9].

#### *β*-TCVAE

The *β*-Total Correlation VAE, or *β*-TCVAE for short, is an extension of the *β*-VAE model. R. Chen et al. showed that decomposing the loss function of *β*-VAE (i.e. Eq 2), and penalizing the total correlation between the latent variables, forces the model to find more statistically independent latent variables [10]. Hence, the loss function becomes:

$$\begin{array}{c} \begin{array}{c} L\left(\theta ,\phi ;x,\alpha ,\beta ,\gamma \right)=-E_{q(z|x)}\left[log\;p\left(x|z\right)\right]+\alpha \;D_{KL}\left(q\left(z,x\right)||q\left(z\right)p\left(x\right)\right)\\ +\beta \;D_{KL}\left(q\left(z\right)||\;\Pi q\left(z\right)\right)+\gamma \;\Sigma D_{KL}\left(q\left(z\right)||p\left(z\right)\right) \end{array} \end{array}$$ (3)

Here, the KL term in Eqs 1 and 2 is decomposed into 3 different terms. The first term, preceded by *α*, is modeling the mutual information between the data variable and latent variables. The second term, preceded by *β*, is modeling the dependence between the different latent variables and is called the total correlation (TC) term. The last term that is preceded by *γ* is used to prevent each individual latent variable from diverging away from its prior. This work showed that penalizing the total correlation term (i.e. setting *β* in Eq 3 to a large positive value) helps the VAE to learn disentangled representations [10]. However, the effect of weighting the three different terms in finding a disentangled latent space is hard to assess.

#### DIP-VAE

Disentangled Inferred Prior VAE (DIP-VAE) learns a disentangled representation by matching the covariance of the prior distribution and the latent distribution. Authors argued that achieving a disentangled representation requires a disentangled prior [11]. The model uses the following loss function:

$$\begin{array}{c} L\left(\theta ,\phi ;x\right)=-E_{q(z|x)}\left[log\;p\left(x|z\right)\right]+D_{KL}\left(q\left(z|x\right)\;||\;p\left(z\right)\right)+\lambda \;D\left(q\left(z\right)||\;p\left(z\right)\right) \end{array}$$ (4)

In the last term, *D*(.), denotes a distance metric between *p*(*z*) and the (intractable) *q*(*z*). The authors modeled this distance as the squared difference between the two corresponding covariance matrices. This means that DIP-VAE minimizes the covariance between latent factors, while *β*-TCVAE minimizes the correlation. This squared difference can in practice be computed in two ways, leading to two sub-variants termed DIP-VAE I and DIP-VAE II (see [11] for more details). Here, we used DIP-VAE II where the latter term is computed as shown in Eq 5:

$$\begin{array}{c} \lambda \;D\left(q\left(z\right)||\;p\left(z\right)\right)=\lambda_{od}\Sigma_{i\neq j}{\left[Cov_{q(z)}\left[z\right]\right]}_{ij}^{2}+\lambda_{d}\Sigma_{i}{\left({\left[Cov_{q(z)}\left[z\right]\right]}_{ii}-1\right)}^{2} \end{array}$$ (5)

The λ_*d*_ and λ_*od*_ variables are used to weigh the contribution of the disentanglement objective.

#### IWAE

Importance Weighted AutoEncoder (IWAE) provided a tighter ELBO of the data log-likelihood compared to the vanilla VAE [3]. This was achieved by drawing *K* samples instead of one from the encoder network in order to perform the Monte Carlo estimate of the expectation term of Eq 1 [23]. Thus the loss function becomes:

$$\begin{array}{c} \hat{L_{K}}=-\text{log}\;\frac{1}{K}\sum_{i=1}^{K}\frac{p(x|z)\;p(z)}{q(z|x)} \end{array}$$ (6)

Note that this loss cannot be decomposed to a reconstruction term and a KL divergence term for *K* > 1.

#### Categorical VAE

Categorical VAE (CAT-VAE) made it possible to model a discrete latent space, unlike vanilla VAE that consider continuous Gaussian latent space. CAT-VAE introduced the Gumbel-softmax distribution that is a continuous distribution that approximates the categorical distribution [22]. CAT-VAE can incorporate label information, but for a fair compression between the different models we did not use the labels of the samples in the training of this model. The loss function of CAT-VAE for unlabeled data is shown in Eq 7, where we marginalize over all possible labels *y*.

$$\begin{array}{c} L\left(\theta ,\phi ;x\right)=E_{z∼q(y,z|x)}\left[q\left(y,z|x\right)-\text{log}\;p\left(x|y,z\right)-\text{log}\;p\left(y\right)-\text{log}\;p\left(z\right)\right] \end{array}$$ (7)

### VAE hyperparameters

We studied the effect of hyperparameters on the training of the aforementioned models. We focused on five types of hyperparameters, whose different settings were explored for every VAE model. We set the *latent dimensions* to be either 10, 20, 30, 50, 100 or 200 factors. For the *learning rate* we used 1e-1, 1e-2, 1e-3, 1e-4, 1e-5 and 1e-6 as step size. For the *initialization of weights* of the encoder and the decoder, we compared the following methods: a standard normal (*N*(0, 1)), Uniform (*U*(0, 1)), Xavier normal, Xavier uniform [25] and Kaming uniform [26]. We compared three *optimizers*: Adam [27], RMSprop [28] and Stochastic Gradient Descent (SGD) [29]. Finally, we tested the effect of two *activation functions* on the neural networks: Rectified linear unit (ReLU) [30, 31] and Hyperbolic tangent (tanh).

### Datasets

The models were trained on the TCGA RNA-seq gene expression dataset [14]. The data were downloaded from [32]. This dataset contains log-transformed counts already filtered by removing the lowly expressed genes, around 10% of the genes and batch corrected using the EB++ algorithm which is a variation of the Empirical Bayes/ComBat algorithm to accommodate for platforms and protocol differences [33]. The samples that have a cancer type label in the meta data were selected [34]. This gave us a total of 11,014 samples from 33 different cancer types. The 5,000 most variable genes across all samples were selected based on the mean absolute deviation (MAD). Each gene was centred and scaled to zero mean and unit variance using z-score normalisation.

To assess the generalizability of the models’ hyperparameters, we retrained all models on the GTEx RNA-seq dataset [35]. We used the gene expression V8 data that were downloaded from [36]. These gene expression data were already batch corrected and were transformed to Transcripts Per Million (TPM) counts. The data comprises 17,382 samples with 56,200 genes representing 30 different tissue types. We followed the same procedure as the TCGA dataset in selecting the top 5000 genes.

### Evaluation of the effect of hyperparameters

The network architecture was held fixed: The encoder and decoder were made from two fully-connected layers as Hu and Greene showed that this design consistently outperformed architectures with different number of layers [37]. The whole design is following the design proposed by Way and Greene [13]. For the encoder, the first layer went from 5000 nodes to 512 nodes, and the second layer went from 512 nodes to the the number of nodes equal to the selected dimension for the latent space. The decoder architecture was the reverse (latent dimension to 512 nodes, and a second layer from 512 nodes to 5000 nodes).

For each of the six VAE variants, we ran an exhaustive grid search to evaluate all possible combinations of latent dimensions, learning rate, optimizer, initialization and activation function, leading to a total of 1080 different setups per VAE variant. To account for variation stemming from the random initialization of the weights and of the data splitting, each setup was trained 10 times and we reported the mean and standard deviation of the final loss. During each run, the TCGA dataset was randomly split into a training (70%) and a validation set (30%) stratified per cancer type. We trained the models on the training data for 1000 epochs and applied early stopping if the validation loss did not improve for longer than 3 epochs. The mean validation loss over the 10 random restarts was used as the criterion for evaluating hyperparameter combinations.

We evaluated the ability of the models to perform both unsupervised and supervised downstream tasks. We used clustering as unsupervised task to cluster the input data in the latent space. To do so, for each hyperparameter combination, the whole dataset was embedded into the latent space (*z*). Then, the embeddings were used to cluster the data using the Leiden community detection algorithm [38]. The neighbourhood graph for the Leiden algorithm was created based on the default settings, using the 15 nearest neighbours found according to the Euclidean distance. Then, the Adjusted Rand Index (ARI) was calculated between the found clusters and the known cancer type labels [39].

For the supervised task, we conducted survival analysis using Cox proportional hazards model [40]. For each hyperparameter configuration, embeddings served as input features for the survival analysis. Age, gender, and cancer type were included as covariates in the analysis. Gender and cancer type were represented as one-hot encoded features. The endpoint of the analysis was the overall survival. To evaluate the relative fit of the different models, we utilized Akaike’s Information Criterion (AIC) [41]. The AIC is calculated using the following equation:

$$\begin{array}{c} AIC=2k-2ln(\hat{L}) \end{array}$$ (8)

Where *k* is the number of estimated parameters by the Cox model, while $\hat{L}$ is the model’s likelihood. The AIC provides a measure that balances model fit and complexity. Lower AIC values indicate a better model fit with less complexity.

After evaluating the impact of various hyperparameters on the TCGA dataset, we conducted an additional experiment to validate and generalize the hyperparameter recommendations derived from our analysis. For this purpose, we used the GTEx dataset and ran all the different configurations for VAE model only once. To ensure proper model assessment, the GTEx dataset is split in a stratified manner by the sample tissue into training and validation sets, allocating 70% and 30% of the samples, respectively.

### Evaluation of disentanglement

We selected the recommended configurations for all VAE models (i.e. learning rate, optimizer, initialization and activation) derived from the hyperparameter evaluations (see Results). To evaluate the impact of the latent dimension size on disentanglement, we retrained all recommended VAE models and repeated the experiments using latent dimension sizes ranging from 10 to 200. We assessed disentanglement based on three different criteria. First, to assess the separability and informativeness of the learned latent variables of these configurations, we employed the Weighted SEParability and INformativeness (WSEPIN) metric [8]. This metric quantifies the extent to which the latent factors (*z*) are separable from one another while also retaining meaningful information about the input data (*x*). The calculation of WSEPIN is based on the following equation:

$$\begin{array}{c} WSEPIN=\sum_{i=0}^{L-1}\rho_{i}I\left(x,z_{i}|z_{\neq i}\right) \end{array}$$ (9)

where

$$\begin{array}{c} \rho_{i}=\frac{I(x,z_{i})}{\sum_{j=0}^{L-1}I\left(x,z_{j}\right)} \end{array}$$ (10)

While *I*(*x*, *z*_*i*_) is the mutual information between *x* and *z*_*i*_. It is worth noting that the higher the value of *I*(*x*, *z*_*i*_|*z*_≠*i*_) the more disentangled *z*_*i*_.

Second, we evaluated the ability of these configurations to encode specific data features of interest, solely using one or two latent variables, by calculating the Spearman correlation between a latent variable and a data feature using Eq 11.

$$\begin{array}{c} \rho_{r_{z},r_{y}}=\frac{Cov(r_{z},r_{y})}{\sigma_{r_{z}}\sigma_{r_{y}}} \end{array}$$ (11)

Where *r*_*z*_ and *r*_*y*_ are the ranked latent variables and data features respectively, while $\sigma_{r_{z}}$ and $\sigma_{r_{y}}$ are the standard deviation of the ranked latent variables and data features respectively. We set the threshold of correlation at *ρ* = 0.1 which is approximately equivalent to the statistical significance threshold for the correlation using an alpha of 0.05.

Data features tested were: age, days to metastasis event, immune infiltration [42], and the presence of either of the mutation signatures SBS 1,2,5,13,15 and 40 determined with exome sequencing [43]. In addition to the three aforementioned features, we also evaluated the disentanglement of gender by calculating its correlation with latent variables using a logit model. We determined how many of these 10 data features were encoded by each model, i.e. how many features are correlated with at least one latent factor. Then we measured whether these features are encoded in a disentangled representation, which we defined as being correlated with only one or two latent factors.

Third, the Robust Mutual Information Gap (RMIG) metric was employed to quantify the interpretability of the latent space factors [8]. This metric allowed us to assess the interpretability of the latent variables in relation to the data features of interest. The calculation of RMIG is based on the following equation:

$$\begin{array}{c} RMIG\left(y_{k}\right)=I\left(z_{i1},y_{k}\right)-I\left(z_{i2},y_{k}\right) \end{array}$$ (12)

where *y*_*k*_ are the data features, while *z*_*i*1_ and *z*_*i*2_ are the factors with the highest and second highest mutual information with *y*_*k*_. To facilitate the interpretation of the RMIG measure, authors normalized it by dividing the RMIG score by the entropy of the corresponding data feature *H*(*y*_*k*_) [8]. This made the normalized RMIG score range from 0 to 1, where the higher the value the more the feature is disentangled. We calculated the normalized RMIG score for each data feature across the different configurations employed in the disentanglement experiment.

## Results

### The validation loss does not always reflect downstream performance

**Fig 2:**
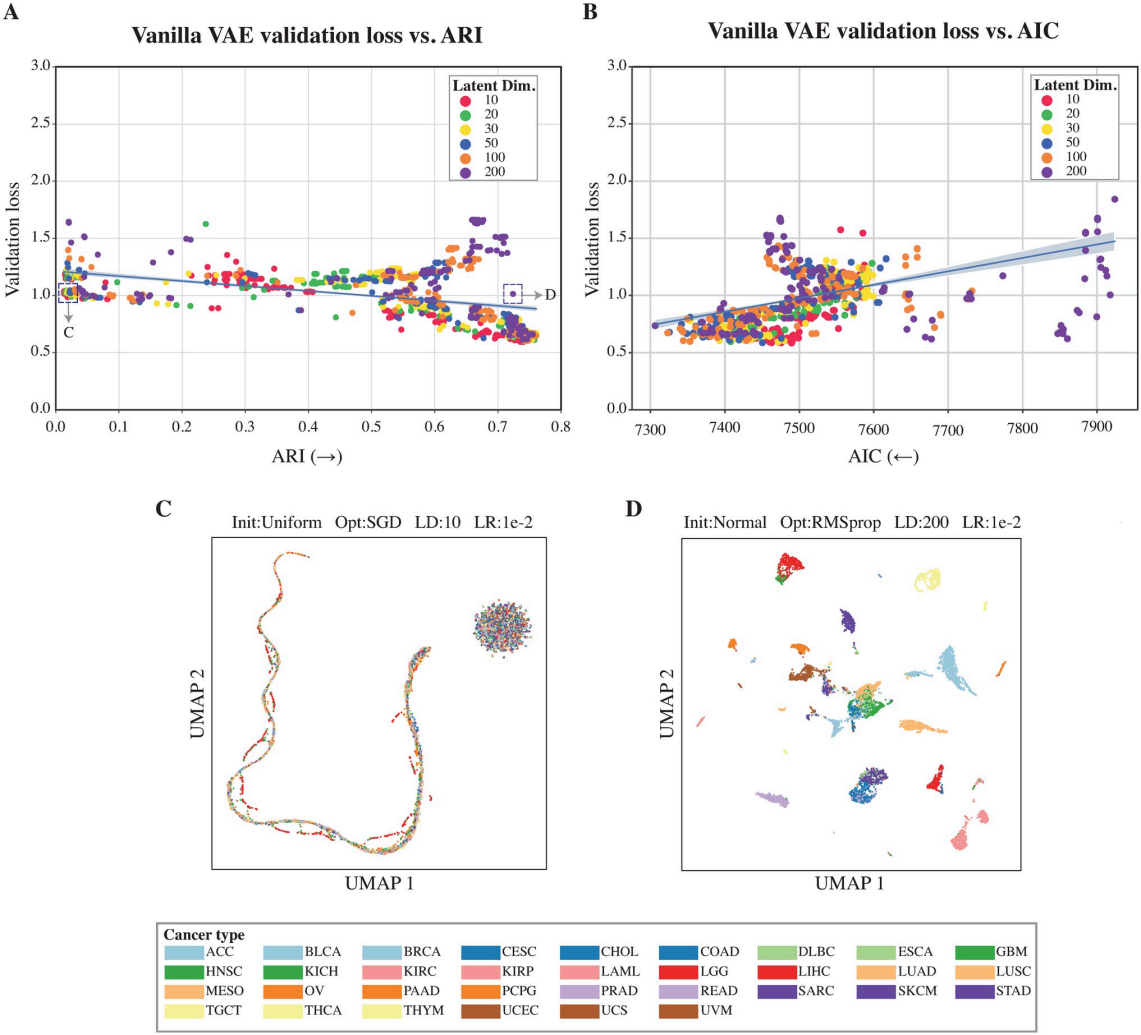
Validation loss does not reflect downstream performance. Plotting the 90th percentile (i.e., excluding the highest 10%) of the of the validation loss (*y*−axis) for the different vanilla VAE hyperparameters configurations vs: A) the ARI (*x*−axis, the higher the better) and B) the AIC (*x*−axis, the lower the better). The figure shows a correlation between the validation loss and ARI & AIC, however, different configurations with the same validation loss can have different scores. The blue line shows the regression line and its thickness indicates the 95% confidence interval. The dots are colored after the latent space dimensions variable. C/D) UMAP visualization of the TCGA data embedded into the learned latent space for a Vanilla VAE configuration. C) For a configuration with a validation loss ≈ 1 and an ARI score ≈ 0 (good model fit, poor clustering ability). D) For a configuration with a validation loss ≈ 1 and an ARI score of ≈ 0.72 (good model fit, and good clustering performance).

We tested the performance of six different VAE models, while varying five different hyperparameters on the TCGA RNA-seq data. To evaluate the learned latent space, we passed all data points through the encoder of each trained model to extract the corresponding embeddings and used them on both unsupervised and supervised downstream tasks. For clustering as an unsupervised task, we clustered the embeddings using the Leiden algorithm [38]. The models were then evaluated on whether their resulting clustering overlaps with the different cancer types using ARI as evaluation measure. Fig 2A and S1–S5 Figs show that despite the correlation between loss and ARI (Spearman |*ρ*| = 0.53 for vanilla VAE, S2 Table), models with the same loss can have different ARI values. For example, Fig 2C & 2D show the learned embeddings of two hyperparameter combinations that had both a loss of value 1. One of the models generates a clustering resulting in an ARI ≈ 0.01 (Fig 2C), while the clustering as a result of the other model has an ARI ≈ 0.72 (Fig 2D).

For the supervised task, we used the latent features learned by each model to fit a Cox proportional hazards model, aiming to predict overall survival. Fig 2B and S1–S5 Figs, show the correlation between the validation loss and the AIC (Spearman |*ρ*| = 0.48 for vanilla VAE, S2 Table). We observed a similar pattern when comparing the validation loss with the ARI, where different models exhibited the same validation loss but distinct AIC values. On the other hand, the supervised and unsupervised performance of different hyperparameter settings was highly concordant (Spearman *ρ* = -0.82) (S6 Fig). Together, these results imply that the validation loss of the VAE does not always reflect the performance of the downstream tasks.

### *β*-TCVAE and DIP-VAE are the best performing models

**Fig 3:**
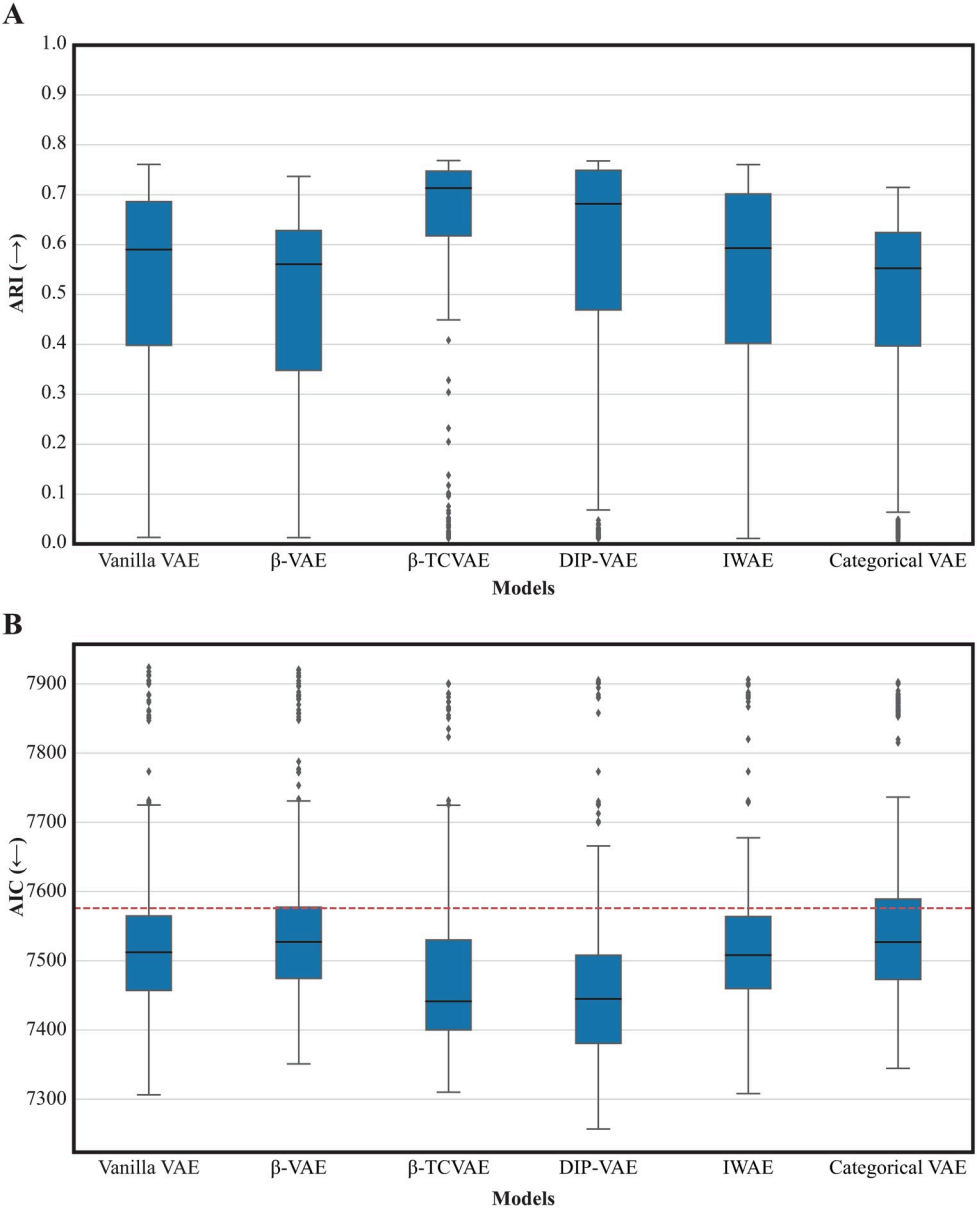
Performance of VAE models in downstream tasks. A) Clustering performance (ARI, *y*−axis, the higher the better) in the latent space of each model (*x*−axis) compared to the true cancer type on the TCGA dataset. Each box represents the distribution of scores obtained for different hyperparameter settings within a specific VAE model. The middle line corresponds to the mean, while the edges of the box represent the first and third quartiles. B) As in A) but for the supervised task of predicting overall survival. Performance is measured by the AIC (*y*−axis, the lower the better) and the dashed red line indicates the baseline model performance using the covariates only (i.e., age, gender and cancer types).

To assess the performance of the different VAE models, we evaluated the scores of the downstream tasks across the different hyperparameter configurations. Overall, the top performance in both unsupervised and supervised tasks was comparable for all VAE models, as shown in Fig 3. This indicates that each of the six models can achieve similar performance when the hyperparameters are properly tuned. However, we observed variations in median performance across models when considering all parameters. We found that *β*-TCVAE and DIP-VAE models performed better than the rest on average in both downstream tasks.

### Choice of hyperparameters affects the VAE performance

**Fig 4:**
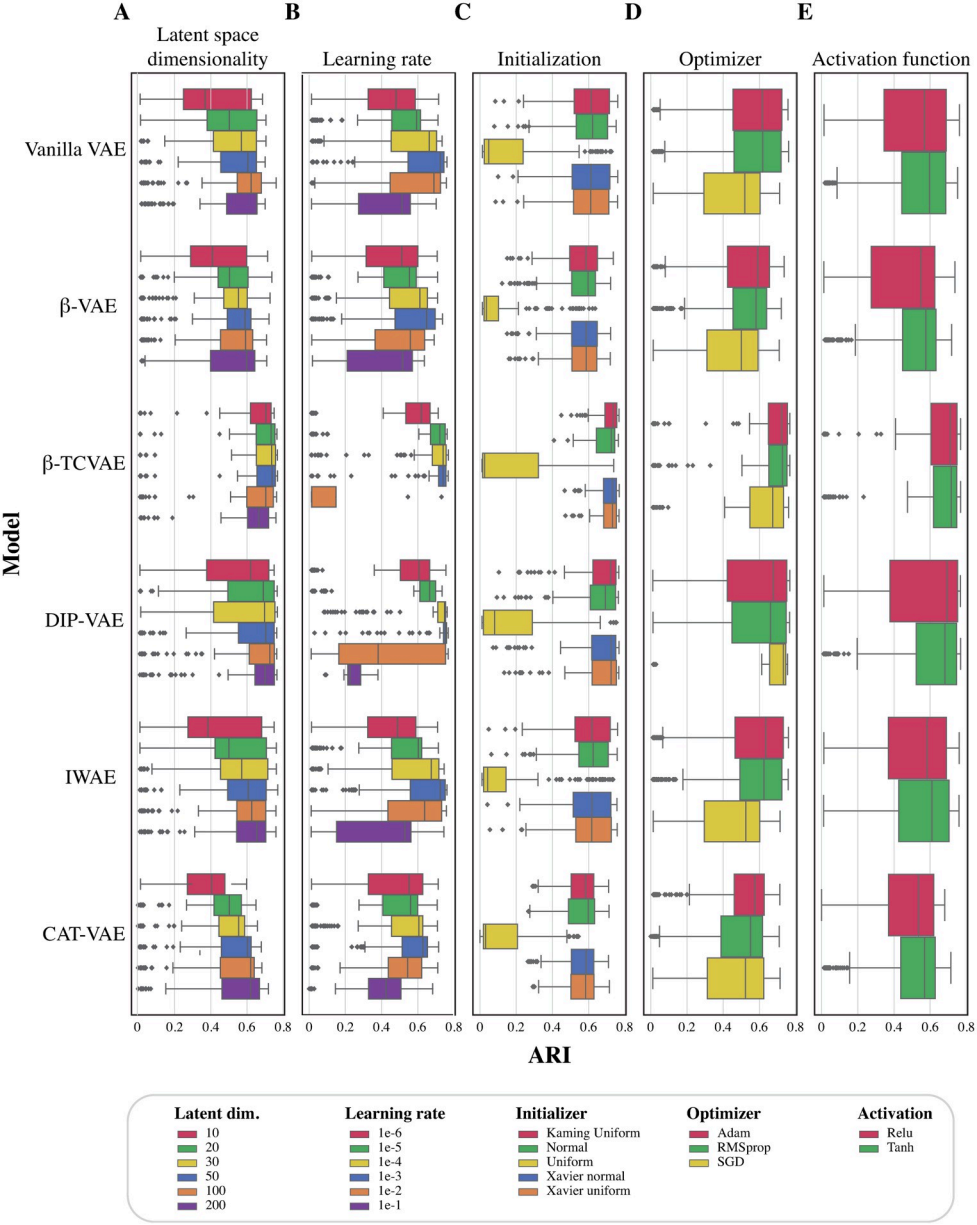
Effect of hyperparameters on different VAE models performance. Each boxplot shows the clustering performance (ARI, *x*−*axis*) of fixing a hyperparameter while varying all others for each VAE model (*y*−axis). The five panels show the five different hyperparameters tested: A) Effect of latent dimensions, B) Effect of learning rate, C) Effect of initialization method, D) Effect of optimizer selection, E) Effect of activation layer.

Next, we tested the effect of each of the five different hyperparameters individually on the performance of different VAE models in terms of the ARI scores. First, we analysed the effect of the number of latent dimensions on the VAE performance (Fig 4A). A small number of latent dimensions compared to the expected number of clusters (i.e. 33 clusters) resulted in lower performances, mid-range values(those that are greater than or equal to the number of clusters, i.e. 50—100) performed well across all models. When examining the effect of the learning rate on the performance, we found that the smallest and the largest learning rates are not performing as well as mid-range learning rates (i.e. 1e-3, 1e-4) see Fig 4B. Moreover, training *β*-TCVAE with a large learning rate failed because the optimization diverged regardless of the choices for the remaining hyperparameters.

In addition, we found that the choice of weight initialization method did not affect performance with the exception of U(0,1) which clearly underperformed the other methods (Fig 4C). For the choice of the optimizer we found that the SGD optimizer on-average results in lower performance, while the Adam optimizer is on average slightly better than RMSprop, see Fig 4D. Finally, for the choice of the activation function we found that the top performance for each activation function is comparable, while on-average tanh gave a slightly better performance (Fig 4E).

**Fig 5:**
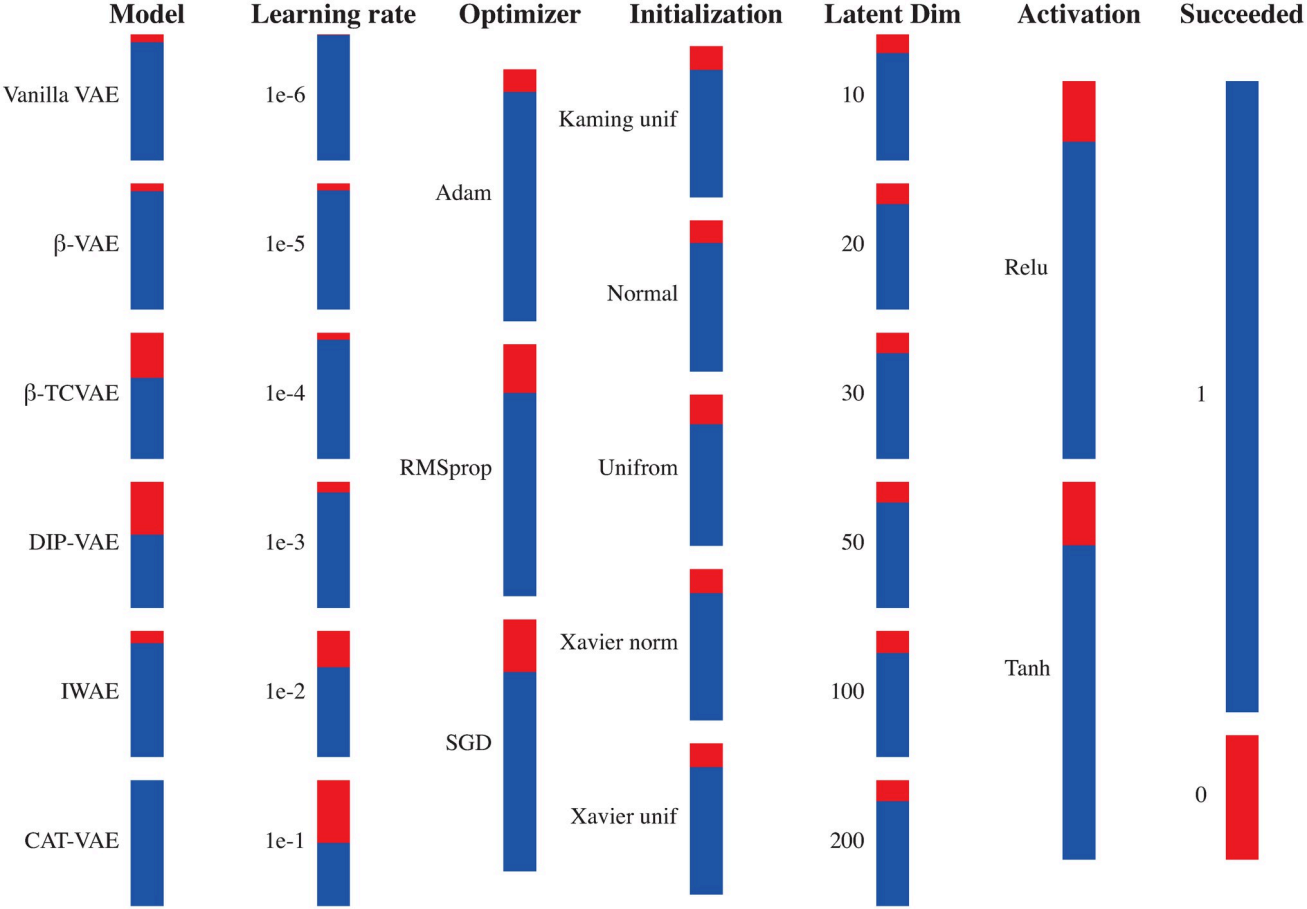
Viability of different hyperparameter combinations for the different VAE models. Columns represent the different hyperparameters. Each bar within a column represents a specific setting of a hyperparameter. The blue color indicates the number of successful configurations, while the red color represents the number of failed configurations. The vertical axis displays the distribution of failed configurations for each specific setting among the 6,480 tested configurations.

Finally, we checked the effect of the five different hyperparameters on the viability of the VAE model configuration, i.e. whether the training managed to converge to a solution or (some of) the weight values diverged to infinity. The main cause of failure is the exploding gradient problem, where the network derivatives are getting very large (i.e. explode) leading to an overflow in network update weights, hence failure in updating weights and training of the network. Fig 5 shows all combinations of the different VAE models and hyperparameters and whether they succeeded or not. We found that most failures are coming from the *β*-TCVAE and DIP-VAE models indicating that these two models are more sensitive to the hyperparameter selection. Contrarily, Categorical VAE never failed in any hyperparameter combination. The learning rate selection is one of the main causes of failure, the smaller the learning rate the less probable the model to fail. The selection of optimizer and initialization method is less crucial to training failure, while the choice of latent dimension size and activation function has minimal impact on the model’s failure rate.

### Selection of hyperparameters generalizes to GTEx dataset

**Fig 6:**
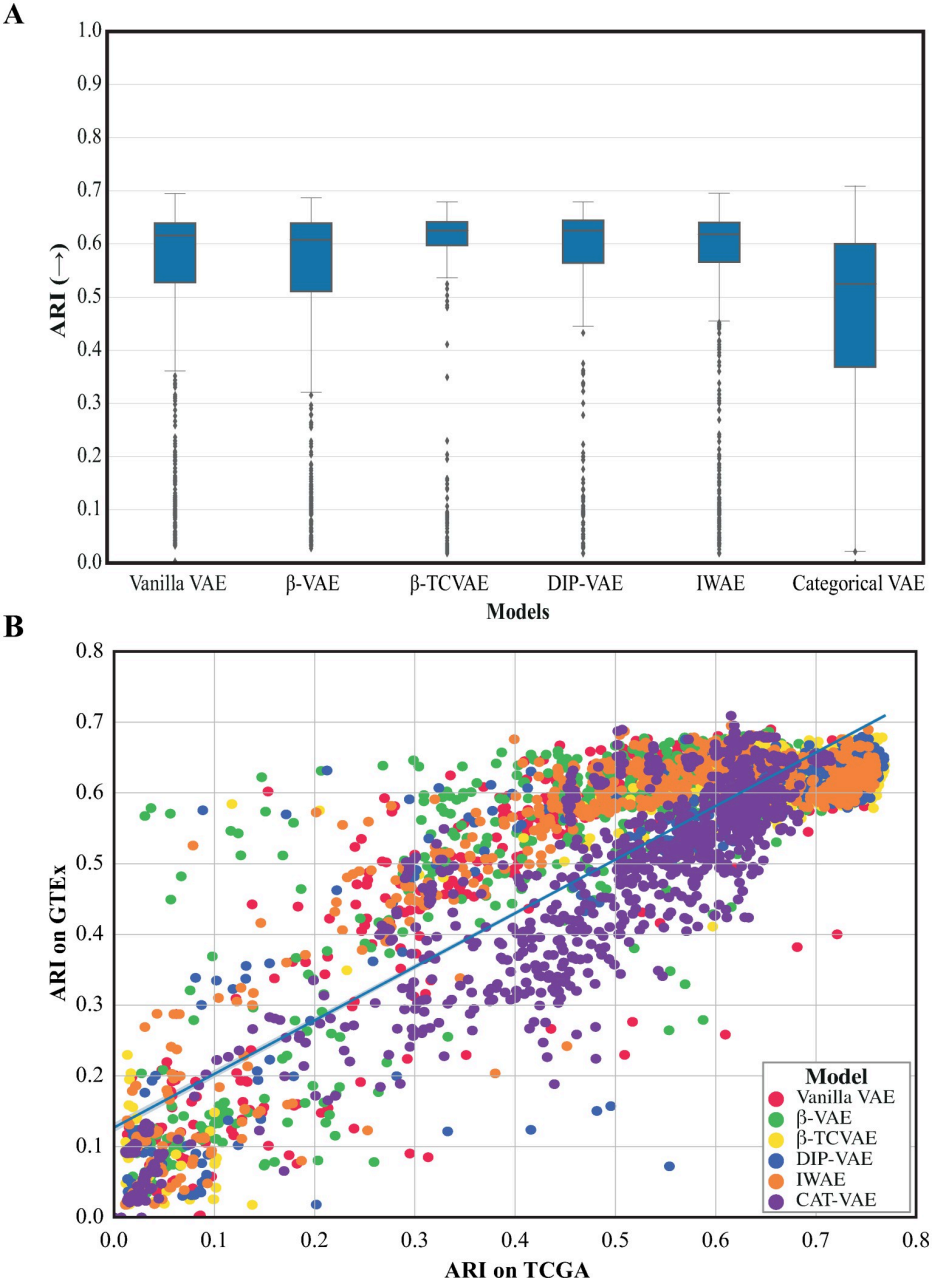
Clustering performance in the GTEx dataset. A) Clustering performance (ARI, *y*−axis, the higher the better) in the latent space of each model (*x*−axis) compared to the known tissue type. Each box represents the distribution of scores obtained for different hyperparameter settings within a specific VAE model. The middle line corresponds to the mean, while the edges of the box represent the first and third quartiles. B) Clustering performance (ARI) between the different VAE configurations in the TCGA (*x*−*axis*) and the GTEx(*y*−*axis*). Dots are colored after the different model and each dot represents a different hyperparameter configuration.

To assess whether the hyperparameters settings associated with good performance on the TCGA data generalize to other datasets, we evaluated their performance on the GTEx dataset. Here we tested whether clustering the samples overlaps with the known tissue types (measured by the ARI). For each VAE model, all configurations were retrained on the GTEx data. The results, presented in Fig 6A, exhibit a clear resemblance to those obtained when testing on the TCGA dataset (Fig 3A). Notably, the *β*-TCVAE and DIP-VAE models consistently outperformed other models on average. Furthermore, we investigated the impact of different hyperparameters (see S7 Fig). The observed effects align with those observed in the TCGA analysis, except for the selection of the optimizer. Interestingly, using the SGD optimizer did not result in a decline in the average performance of the VAEs in the GTEx dataset. To assess the concordance between models performance on TCGA and GTEx, we plotted the ARI scores obtained on TCGA against those obtained on GTEx (Fig 6B). The effect of hyperparameters on clustering performance is significantly correlated between both datasets with *ρ* = 0.7. The consistent patterns observed across diverse datasets provide compelling evidence supporting the generalizability of the hyperparameter recommendations for RNA-seq datasets.

### Latent space disentanglement is not trivial to achieve in an unsupervised manner

Based on our benchmark results, we selected the recommended configuration for training VAE models on this dataset. Hereto we used 1e-3 for learning rate, Kaming uniform initialization, Adam as the optimizer and tanh as the activation function. Based on the proposed definitions of disentanglement, the results are highly dependent on the size of the latent dimension, thus we tested different latent dimension sizes with the aforementioned configuration.

**Fig 7:**
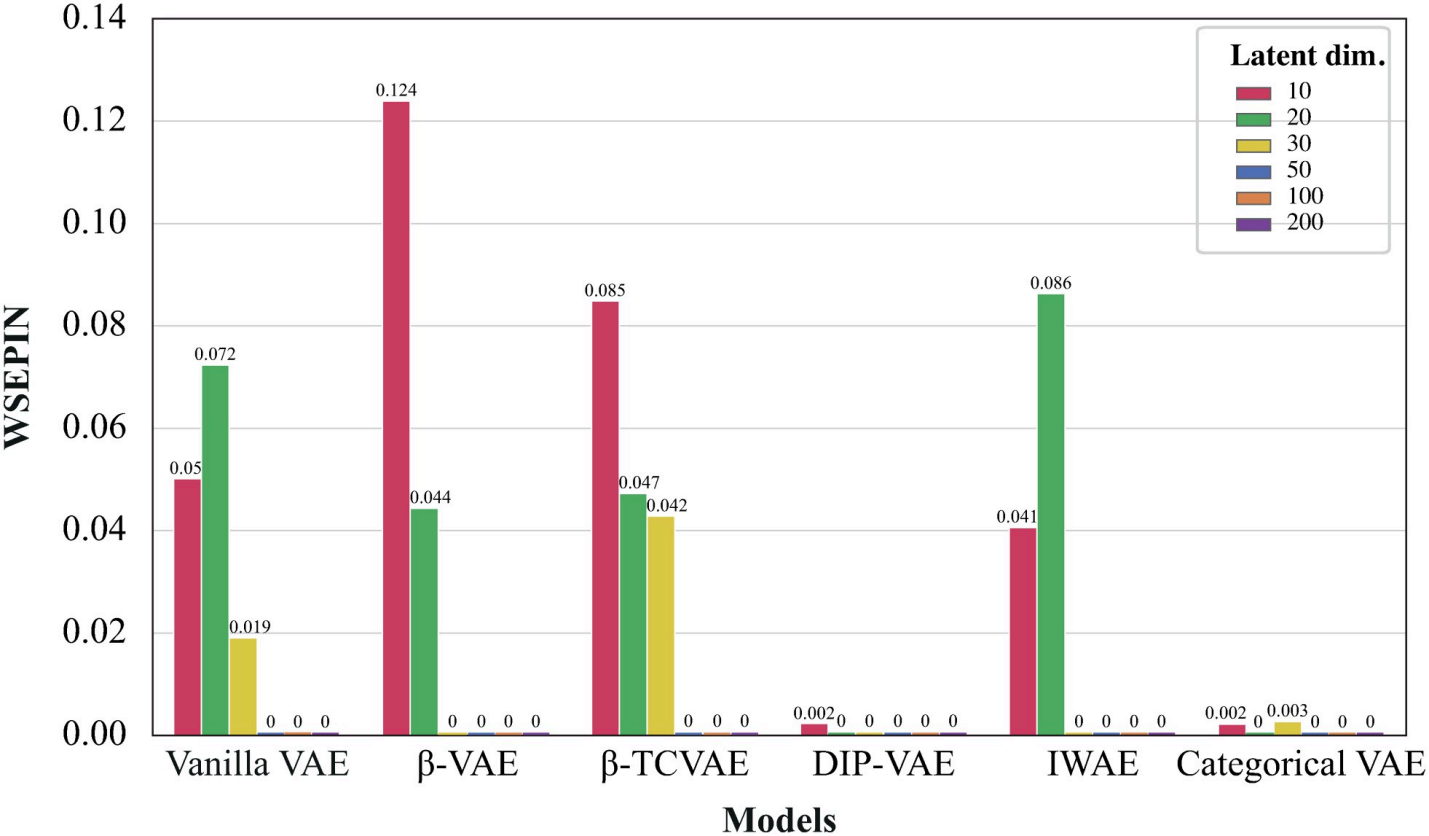
Comparison of VAE models with varying latent dimension sizes based on the WSEPIN metric. The figure shows the WSEPIN score on the *y*−*axis*, while the bars are colored after the different latent dimensions. Numbers over each bar is the approximated WSEPIN score achieved.

First, we assessed the separability and informativeness of the learned latent variables by computing the WSEPIN metric for all the recommended VAE configurations, Fig 7. We observed that the majority of models achieved low WSEPIN scores when the latent dimension varied between 10 and 30. Interestingly, all models achieved zero WSEPIN with latent dimension sizes ranging from 50 to 200. Notably, DIP-VAE and CAT-VAE consistently achieved the lowest scores across different configurations, while the best WSEPIN achieved with *β*-VAE when configured with 10 latent dimensions.

**Fig 8:**
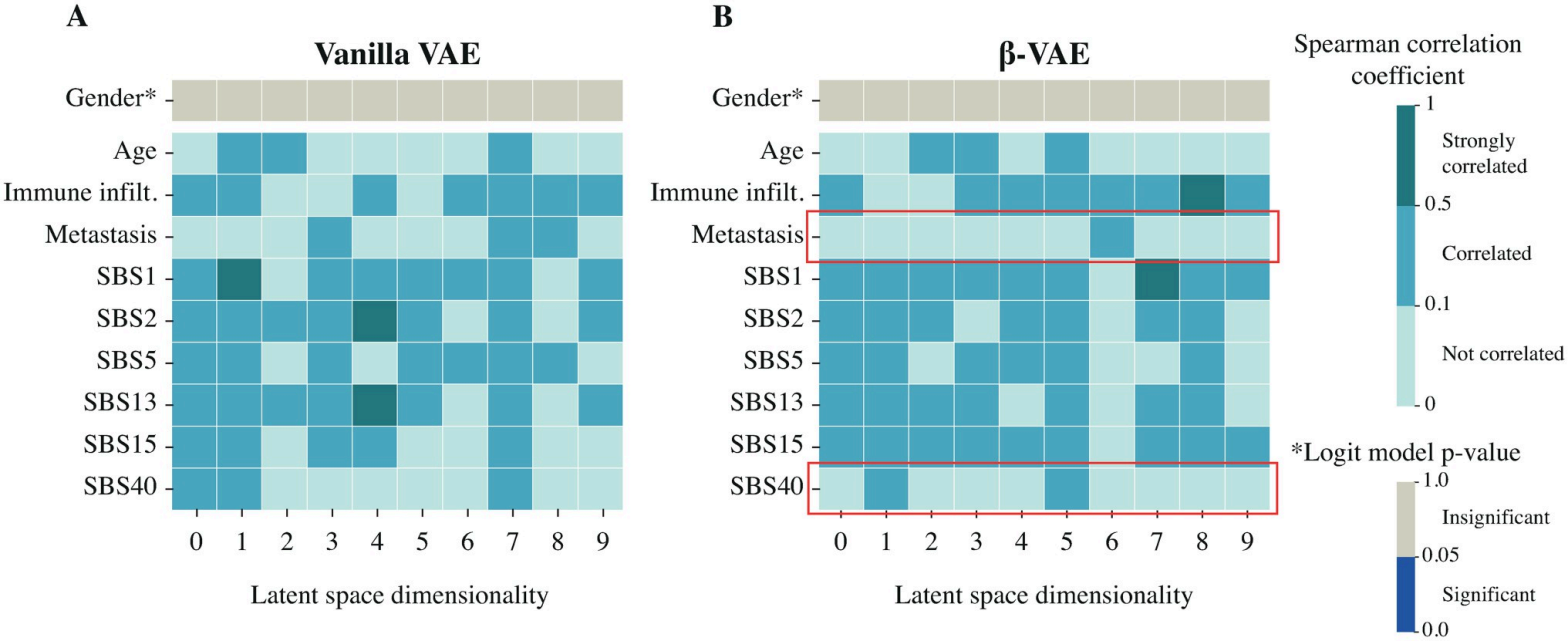
Heatmap showing the Spearman correlation of the latent variables from a 10-dimensional latent space for the vanilla VAE and *β*-VAE with the features. The red highlighting boxes show the disentangled features achieved by a model. A) Vanilla VAE could not disentangle any feature. B) *β*-VAE disentangled metastasis and SBS40.

Next, we calculated the Spearman correlation for each latent space factor of the vanilla VAE and *β*-VAE trained with 10 latent dimensions to each of the data features individually, Fig 8. The vanilla VAE exhibits correlations between all data features and latent space factors, except for gender, where no significant correlation was found using the logit model. However, none of these features were disentangled using the vanilla VAE. Notably, latent space factor 7 showed correlations with all data features.

Analogously, the *β*-VAE resulted in a correlation also between all data features but two of these features were disentangled (Fig 8B). Days to metastasis is correlated with latent space factor 7 solely. SBS40 is correlated with latent space factor 1 and 5 only. Summarizing the *β*-VAE with 10 latent dimensions disentangled 2 data features.

Table 1 shows the disentanglement performances for each VAE model with different latent dimension sizes. The results show that the smaller the latent dimension the better the performance in the disentanglement task. As the number of latent dimensions used for VAE’s increases, more latent space factors start to correlate with the same feature, impacting a model’s disentanglement performance. However, some models can perform better than others for the same latent dimension size. For example, the *β*-VAE and DIP-VAE models that use 10 latent dimensions show the highest number of disentangled latent factors. Only the IWAE model achieved data feature disentanglement when using 200 dimensions for the latent space.

**Table 1:** Number of disentangled data features for different VAE models having different latent dimensions.

Latent Dim.	10	20	30	50	100	200
Model						
Vanilla VAE	0	1	0	0	0	0
*β*-VAE	2	1	0	0	0	0
*β*-TCVAE	2	1	1	1	0	0
DIP-VAE	1	0	1	0	0	0
IWAE	2	1	1	1	0	0
CAT-VAE	1	1	0	0	0	0

**Fig 9:**
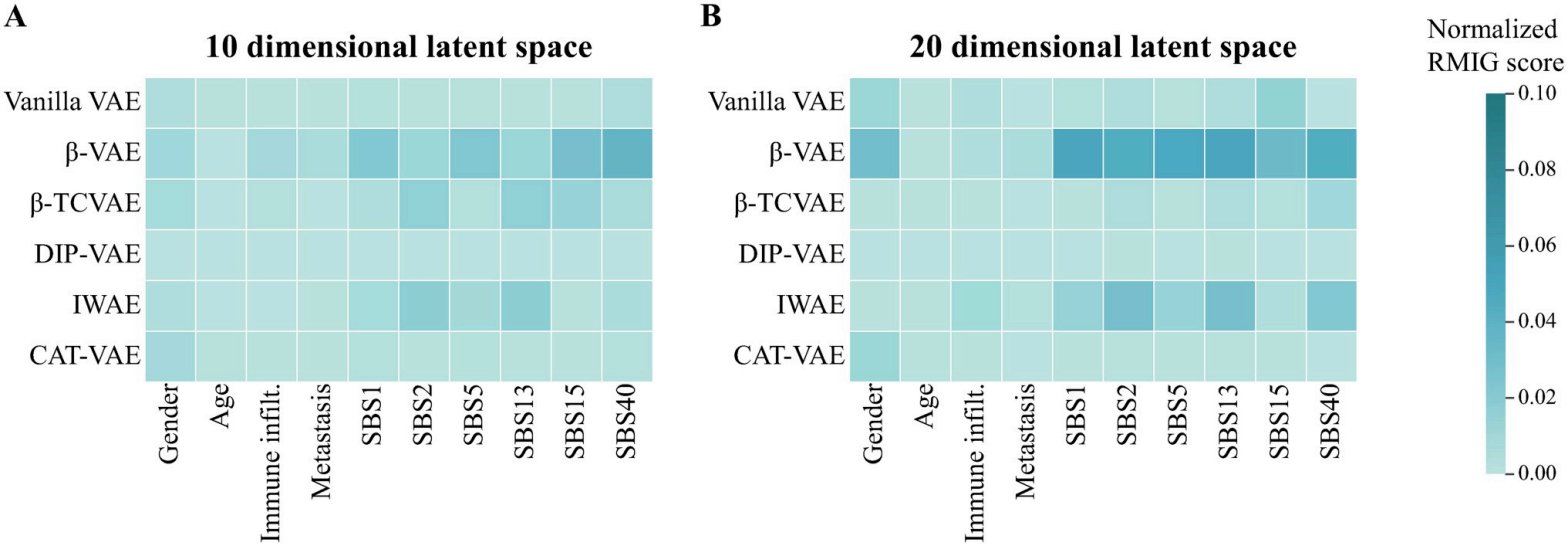
Heatmap showing the normalized RMIG score calculated for different VAE models on data features. A) Using 10 dimensional latent space, *β*-VAE can learn a more disentangled representation compared to other models. B) Using 20 dimensional latent space, *β*VAE and IWAE could disentangle some features compared to other models.

Finally, we evaluated the interpretability of the latent space using the normalized RMIG metric with respect to the data features. Fig 9A displays the normalized RMIG score for the models when using a 10-dimensional latent space. Generally, all models achieved a low normalized RMIG score, which indicates that none of the latent factors uniquely capture information about data features. *β*-VAE could learn a disentangled representation for SBS40, which confirms the results we showed earlier using the Spearman correlation. Similarly, for the 20-dimensional latent space configuration (Fig 9B). Although *β*-VAE and IWAE learn a more disentangled representation for the data features compared to other models, it should be noted that all the scores remained below 0.1 on the normalized RMIG scale, indicating that the features are not completely disentangled. This observation is further supported by calculating the normalized RMIG scores for the 50–200 dimensional latent space, as shown in S8 Fig. Notably, these results confirm our previous findings; that VAE models could not correlate with gender when using a 10 dimensional latent space, while vanilla VAE, *β*-VAE and CAT-VAE can barely disentangle gender when using 20 dimensional latent space.

From these results we conclude that the disentanglement task is in general difficult for all VAE models and that when selecting the latent dimension size there is a trade-off between disentanglement and downstream performance.

## Discussion

This paper studies settings of different VAE models when applied to cluster cancer patients from their RNAseq profile. We found that the validation loss is not always reflective of the performance on downstream tasks that uses the latent space embeddings. Nevertheless, we showed that all VAE variants have the ability to learn a representation of the data that facilitated the downstream tasks either of clustering cancer patients or predicting the overall survival. Despite the fact that *β*-TCVAE and DIP-VAE models had an on-average better performance than others, we can not conclude that they outperform the other models, as all the models could reach a comparable performance based on specific hyperparameter settings. Also, the viability of these two models is too sensitive and susceptible to the hyperparameter selection.

There are multiple possible reasons for the observed inconsistency between the validation loss and the downstream tasks performance. One of them could be the usage of mean square error (MSE) as reconstruction loss, which overemphasizes the effect of outlier samples. These outlier samples could be due to personal/biological differences or technical ones. RNA-seq technologies suffer from different types of technical noise and artifacts [44], that means some samples could be distorted. These distorted samples do not belong to the actual manifold of the data. Then, the squaring factor in the MSE magnifies these errors, making the VAE tries to adopt to these distorted samples. One potential approach to mitigate the impact of MSE is to exert more efforts in filtering the samples, excluding all unwanted heterogeneities [45].

Moreover, we evaluate the mean squared error independently for each gene, without taking gene-gene correlations into account. Although this is a standard practice in the literature, explicitly modelling these correlations might lead to a more meaningful evaluation of reconstruction error. Of course, this would significantly increase the complexity and training time of the model, especially for high-dimensional datasets. Also, we think that using a reconstruction loss function that is less susceptible to outliers as Huber loss [46, 47] or quantile loss [48], will help in better approximating the true manifold.

Another possible explanation could be posterior collapse, a common issue with VAE training [49]. Posterior collapse occurs when the posterior distribution of one or more of the latent variables (*q*(*z*|*x*)) becomes equal to its prior (*p*(*z*)). In other words, the encoder output is random and does not depend on the input sample, so that the collapsed latent features do not encode any meaningful information about the input. When this occurs during training, a flexible-enough decoder can still learn to (partially) reconstruct the input by ignoring the collapsed latent features and/or by overfitting to the encoder’s output. This leads to a relatively low reconstruction loss, despite the fact that the latent features are not a meaningful representation of the data.

One of the main contributions of this paper is that we can provide recommendations on hyperparameters settings when dealing with bulk RNA data. These recommendations are driven from the TCGA dataset and confirmed on the GTEx dataset. Our results show that the selection of hyperparameters greatly influences the performance of the VAE, although this might not be surprising. Considering the expected number of clusters for each dataset, we set the number of latent dimensions to be greater than or equal to the number of expected clusters. For the datasets investigated in this study, we recommend using a latent space of 50—100 dimensions. This recommended range strikes a balance between capturing the complexity of the data and mitigating the risks of overfitting or underrepresentation. Nevertheless, we found that learning disentangled representations in an unsupervised manner is very hard when using those many latent factors and a smaller latent dimension size is preferred if interpretation is important. For the learning rate, we recommend using learning rates between 1e-3 and 1e-4. Large learning rates push a model over the optimum resulting in an oscillating behavior or even make the training fail, whereas low learning rates slow down the learning process tremendously and can get more easily stuck in local minima. For the initialization methods, the uniform methods are not favorable in the deep learning field as the gradient is the same for many nodes, which makes it hard during the training for weight update [50, 51]. The other weight initialization strategies that we tested, included Kaming uniform (default in PyTorch) and Xavier normal (default in Keras) initialization distributions, all these settings resulted in a comparable performance. The SGD optimizer is found to underperform in the other settings on the TCGA dataset. The Adam optimizer is becoming a *de facto* standard, and is widely used in the deep learning, as it is faster and requires less memory to run. Our results show that the Adam optimizer outperforms SGD, and does slightly better than RMSprop. These results are in line with the results by Kingma and Ba for image data [27]. The usage of tanh as the activation function demonstrated, on average, better performance compared to using ReLU, although the top achieved performances were comparable. However, it is important to note that in deeper networks, the usage of ReLU has been shown to be more favorable due to its reduced susceptibility to gradient vanishing and its ability to yield improved performance [31, 52, 53]. In our work, which employed a relatively shallow network architecture with only two layers for the encoder and decoder, we did not encounter the issues typically associated with using the tanh activation function.

Interpretation of machine learning and deep learning models is crucial for their eventual adoption into clinical practice, but this still remains challenging. If (some of) the learned latent factors directly correspond to specific interesting aspects of the data, such as biological processes or important covariates, it would improve the interpretability and therefore the value and potential usage of the VAE models. Our experiments in measuring the disentanglement of the latent factors showed that all VAE models only moderately capture the characteristics tested in the TCGA dataset and that disentanglement often comes at the cost of less good clustering in z-space. Surprisingly, even models specifically designed for learning disentangled representations showed limitations in achieving full disentanglement. The various metrics used in our study consistently indicated that none of the models achieved decent disentanglement of the tested features, as evidenced by low WESPIN scores (less than 0.15) and low normalized RMIG scores (less than 0.1). The correlation plots demonstrated that the VAEs learned complex and entangled representations of the data, which contributed to their performance in downstream tasks but hindered their ability to encode or disentangle specific features. In our experiments, VAE models were able to correlate the same latent factors for both SBS2 and SBS13 which are known to occur in the same samples. These mutational signatures are connected to the activity of the AID/APOBEC family of cytidine deaminases and the activity of the APOBEC enzyme [54, 55]. Again our results align with the theoretical proof of the impossibility of achieving complete disentanglement with completely unsupervised learning [6].

Although the promise of disentanglement with VAEs seems unfulfilled, there are three promising alternatives to force models to learn disentangled representations. The first uses semi-supervised VAE models, where known values of the factors to be disentangled are used to guide the VAE training [56, 57]. The second, stemming from computational neuroscience, imposes biologically-inspired constraints on the weights that enhance selectivity of neurons thereby leading to disentanglement [58, 59]. The third approach relies on the existence of another observed variable, which can be harvested to transform the VAE into non-linear Independent Component Analysis [60]. For example, for the TCGA data this additional variable can be the mutation or methylation profiles of the tumor samples. Further research is needed to validate the utility of these ideas on -omics data.

One limitation of our study is that not all hyperparameters combinations were tested for all models. The effect of hyperparameters weighting the disentanglement terms differs between the different VAE models. We did not study the effect of these hyperparameters on the downstream task as well as the disentanglement task. We decided not to do so because this would result in a unfair (unsystematic) comparison between models. Yet, an important hyperparameter, the number of nodes in a hidden layer, we also did not further explore merely because this would increase the space of models immensely.

In conclusion, we benchmarked several VAE variants on transcriptomics data and studied their learned latent spaces in terms of downstream tasks and disentanglement. Despite a general difficulty to achieve good disentanglement, we found that *β*-TCVAE and DIP-VAE tend to perform best in both tasks, although their training can more easily become unstable when using inappropriate hyperparameters.

## Supporting information

S1 Fig *β*-VAE validation loss vs downstream tasks performance. Scatter plot for the 90th percentile of the validation loss of different hyperparameters configurations of *β*-VAE vs A) ARI, B) AIC. Each dot is a different configuration, and they are colored after the latent space dimensions variable.
(TIF)

S2 Fig *β*-TCVAE validation loss vs downstream tasks performance. Scatter plot for the 90th percentile of the validation loss of different hyperparameters configurations of *β*-TCVAE vs A)ARI, B) AIC. Each dot is a different configuration, and they are colored after the latent space dimensions variable.
(TIF)

S3 Fig DIP-VAE validation loss vs downstream tasks performance. Scatter plot for the 85th percentile of the validation loss of different hyperparameters configurations of DIP-VAE vs A)ARI, B) AIC. The selection of the 85th percentile was motivated by the observation that this particular model tends to generate a higher number of outliers compared to others. Each dot is a different configuration, and they are colored after the latent space dimensions variable.
(TIF)

S4 Fig IWAE validation loss vs downstream tasks performance. Scatter plot for the 90th percentile of the validation loss of different hyperparameters configurations of IWAE vs A) ARI, B)AIC. Each dot is a different configuration, and they are colored after the latent space dimensions variable.
(TIF)

S5 Fig CAT-VAE validation loss vs downstream tasks. Scatter plot for the 90th percentile of the validation loss of different hyperparameters configurations of CAT-VAE vs A)ARI, B)AIC. Each dot is a different configuration, and they are colored after the latent space dimensions variable.
(TIF)

S6 Fig Vanilla VAE downstream tasks performance agreement. Scatter plot for the clustering performance measured in ARI (*y*−*axis*, the higher the better) and survival analysis performance measured in AIC (*x*−*axis*, the lower the better). The figure demonstrates the concordance between these two measures, indicating that models with higher ARI tend to have lower AIC. The top left quarter of the plot represents the best performing models across both clustering and survival analysis tasks. Blue line represents the lowess curve fitting for the data.
(TIF)

S7 Fig Effect of hyperparameters on different VAE models measured on the GTEx dataset. Each boxplot shows the clustering performance (ARI, *x*−*axis*) of fixing a hyperparameter while varying all others for each VAE model (*y*−axis). The five panels show the five different hyperparameters tested: A) Effect of latent dimensions, B) Effect of learning rate, C) Effect of initialization method, D) Effect of optimizer selection, E) Effect of activation layer. The figure shows analogous effect to that found on TCGA dataset.
(TIF)

S8 Fig Heatmap showing the normalized RMIG score calculated for different VAE models on data features. A) Using 30 dimensional latent space. B) Using 50 dimensional latent space. C) Using 100 dimensional latent space. D) Using 200 dimensional latent space.
(TIF)

S1 Table VAE models hyperparameters. A listing of the hyperparameters that were held constant throughout the study. The values were set according to the implementation of https://github.com/AntixK/PyTorch-VAE.
(PDF)

S2 Table Spearman correlation between different models validation loss and ARI, AIC. The absolute rounded Spearman correlation between all the different configurations tested for each model and both ARI and AIC values achieved by this model in the downstream task.
(PDF)
