# Supplementary figures and images for: Benchmarking variational AutoEncoders on cancer transcriptomics data
Source: PLoS One. 2023 Oct 5;18(10):e0292126. doi: 10.1371/journal.pone.0292126 (PMC10553230; doi:10.1371/journal.pone.0292126)

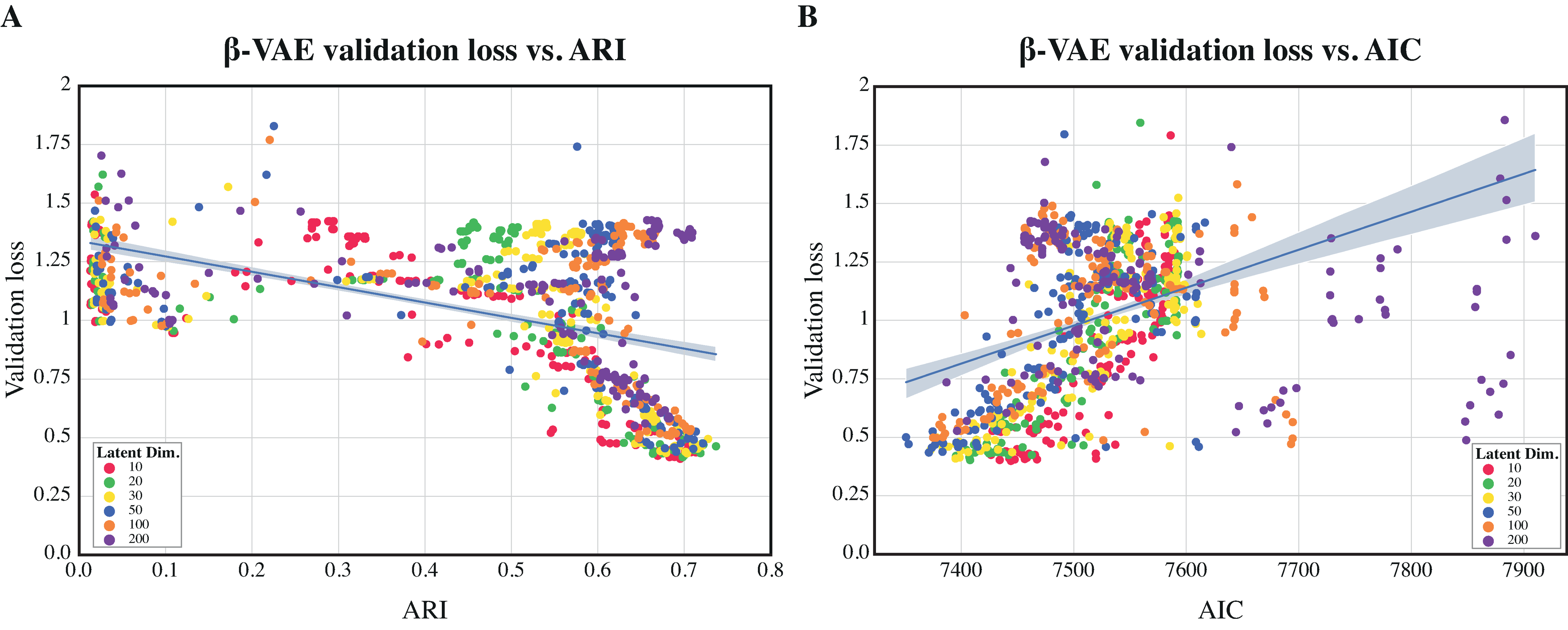

Supplement: S1 Fig — Scatter plot for the 90th percentile of the validation loss of different hyperparameters configurations of β-VAE vs A) ARI, B) AIC. Each dot is a different configuration, and they are colored after the latent space dimensions variable. (TIF) [file pone.0292126.s001.tif]

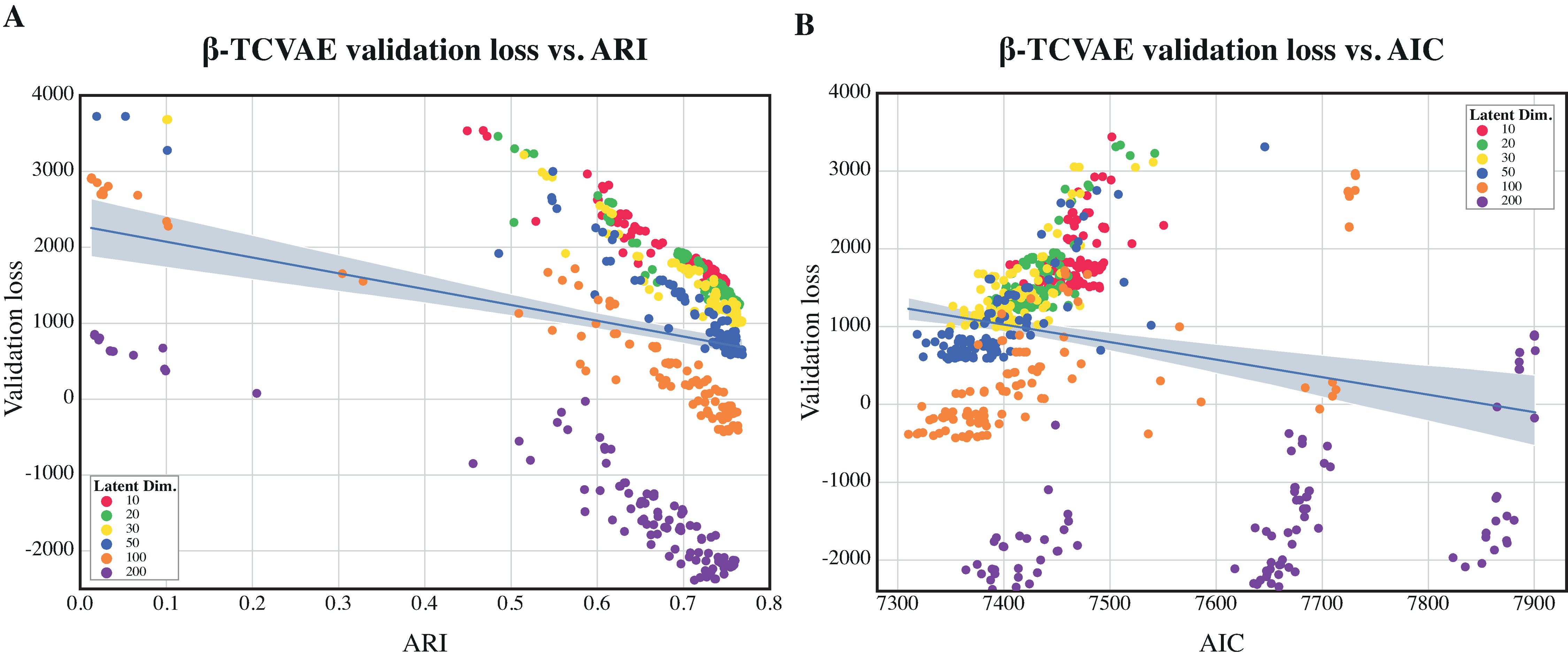

Supplement: S2 Fig — Scatter plot for the 90th percentile of the validation loss of different hyperparameters configurations of β-TCVAE vs A)ARI, B) AIC. Each dot is a different configuration, and they are colored after the latent space dimensions variable. (TIF) [file pone.0292126.s002.tif]

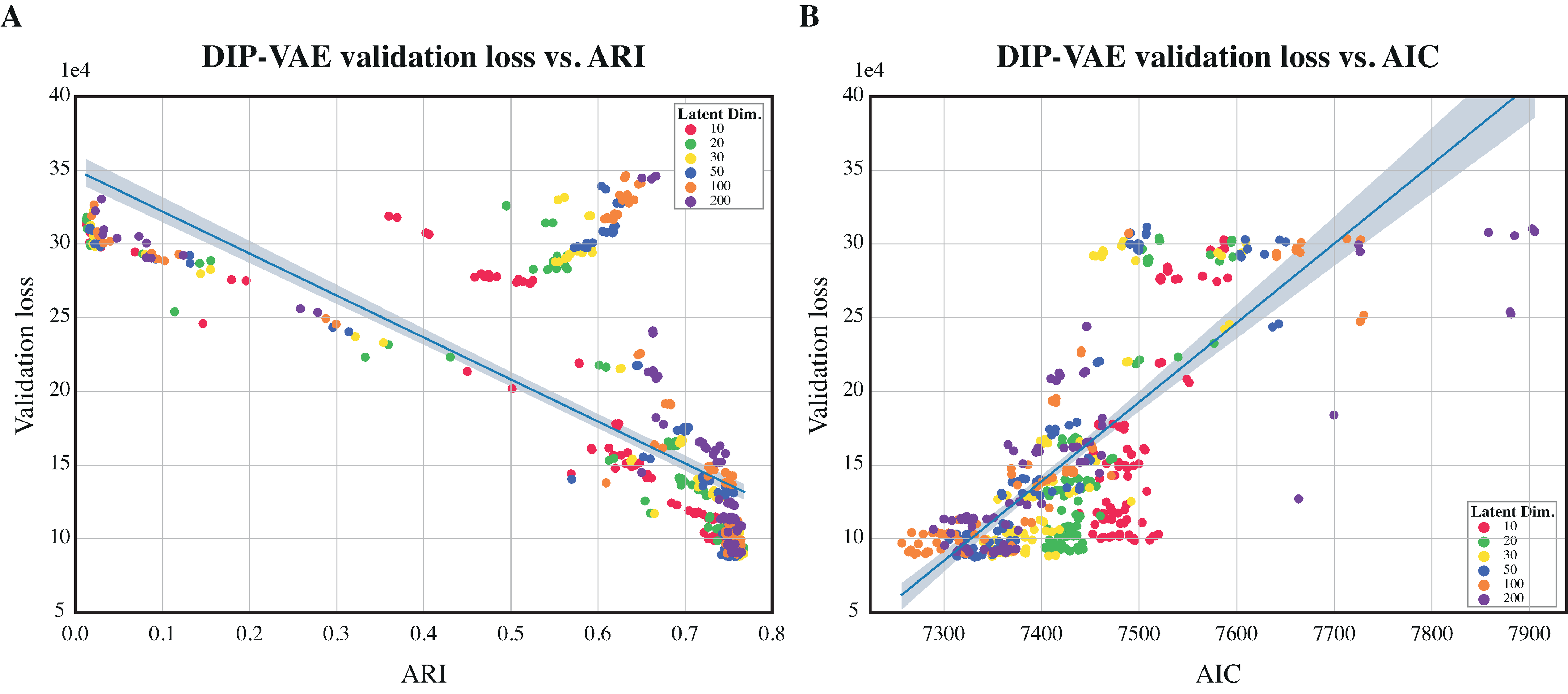

Supplement: S3 Fig — Scatter plot for the 85th percentile of the validation loss of different hyperparameters configurations of DIP-VAE vs A)ARI, B) AIC. The selection of the 85th percentile was motivated by the observation that this particular model tends to generate a higher number of outliers compared to others. Each dot is a different configuration, and they are colored after the latent space dimensions variable. (TIF) [file pone.0292126.s003.tif]

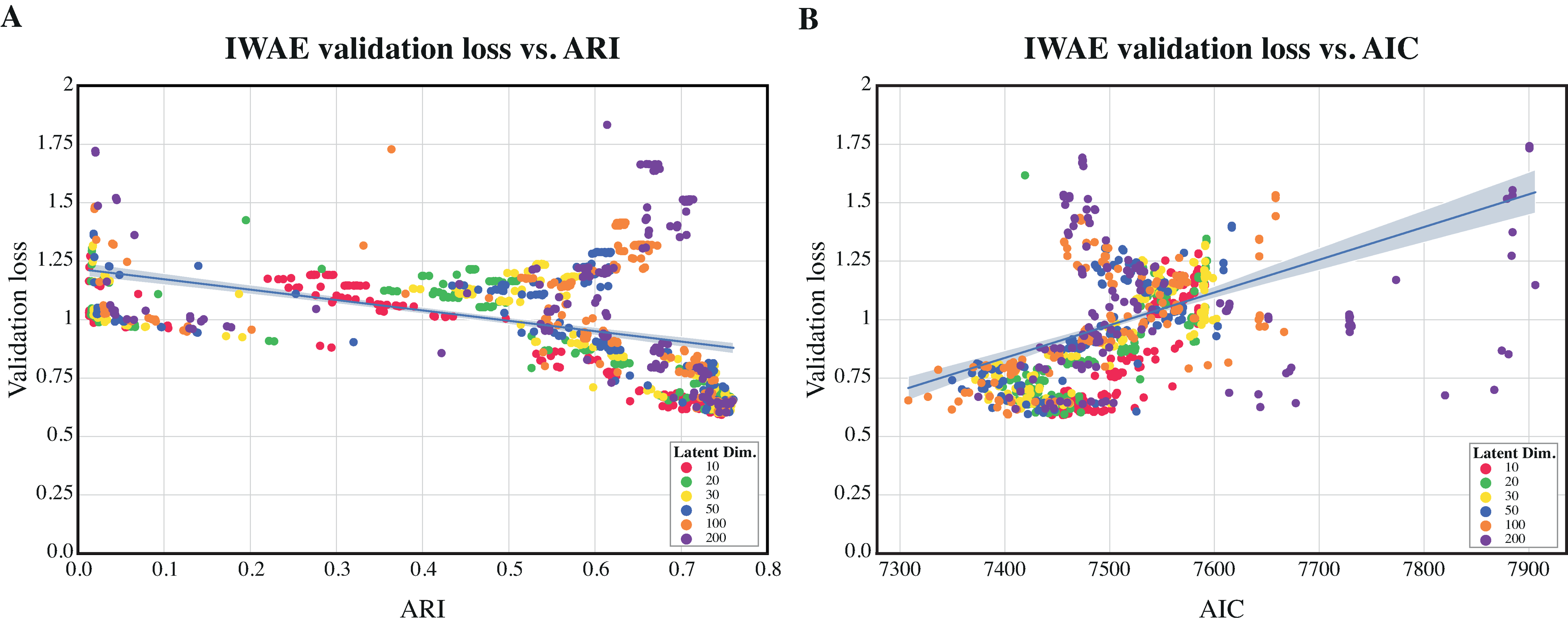

Supplement: S4 Fig — Scatter plot for the 90th percentile of the validation loss of different hyperparameters configurations of IWAE vs A) ARI, B)AIC. Each dot is a different configuration, and they are colored after the latent space dimensions variable. (TIF) [file pone.0292126.s004.tif]

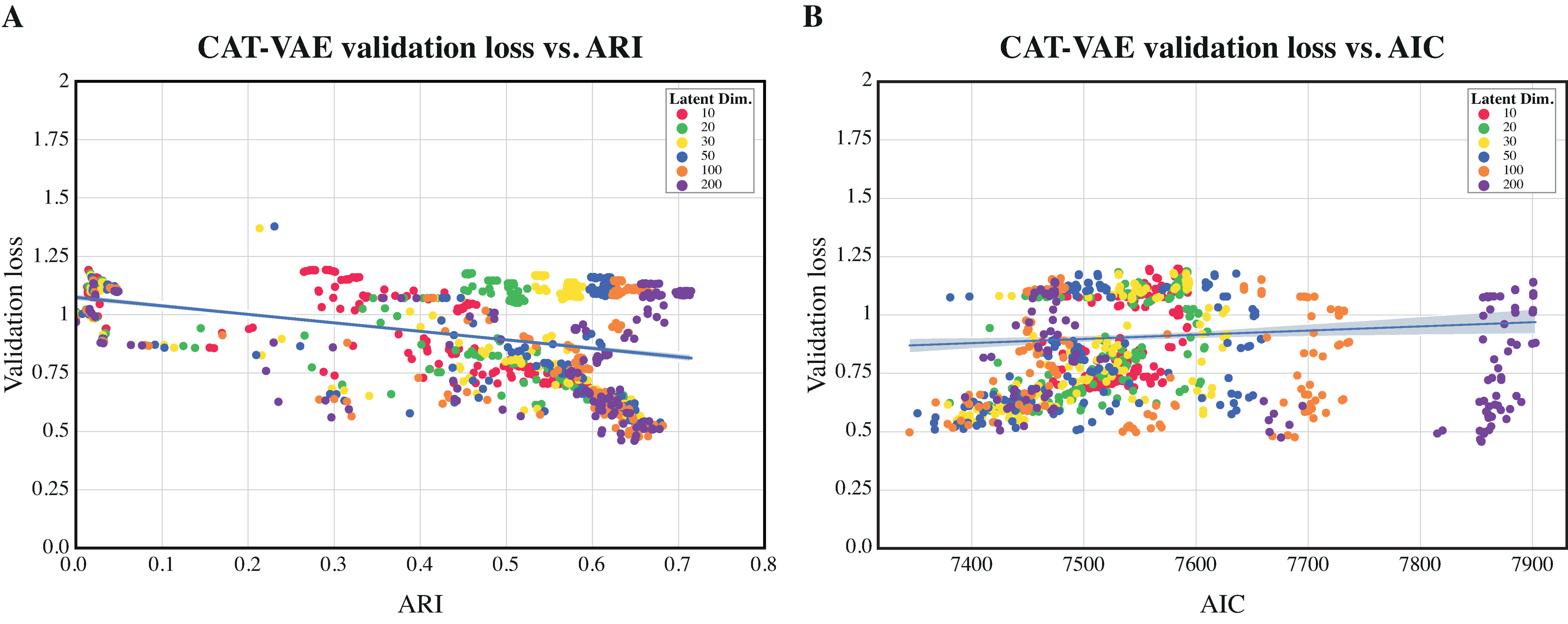

Supplement: S5 Fig — Scatter plot for the 90th percentile of the validation loss of different hyperparameters configurations of CAT-VAE vs A)ARI, B)AIC. Each dot is a different configuration, and they are colored after the latent space dimensions variable. (TIF) [file pone.0292126.s005.tif]

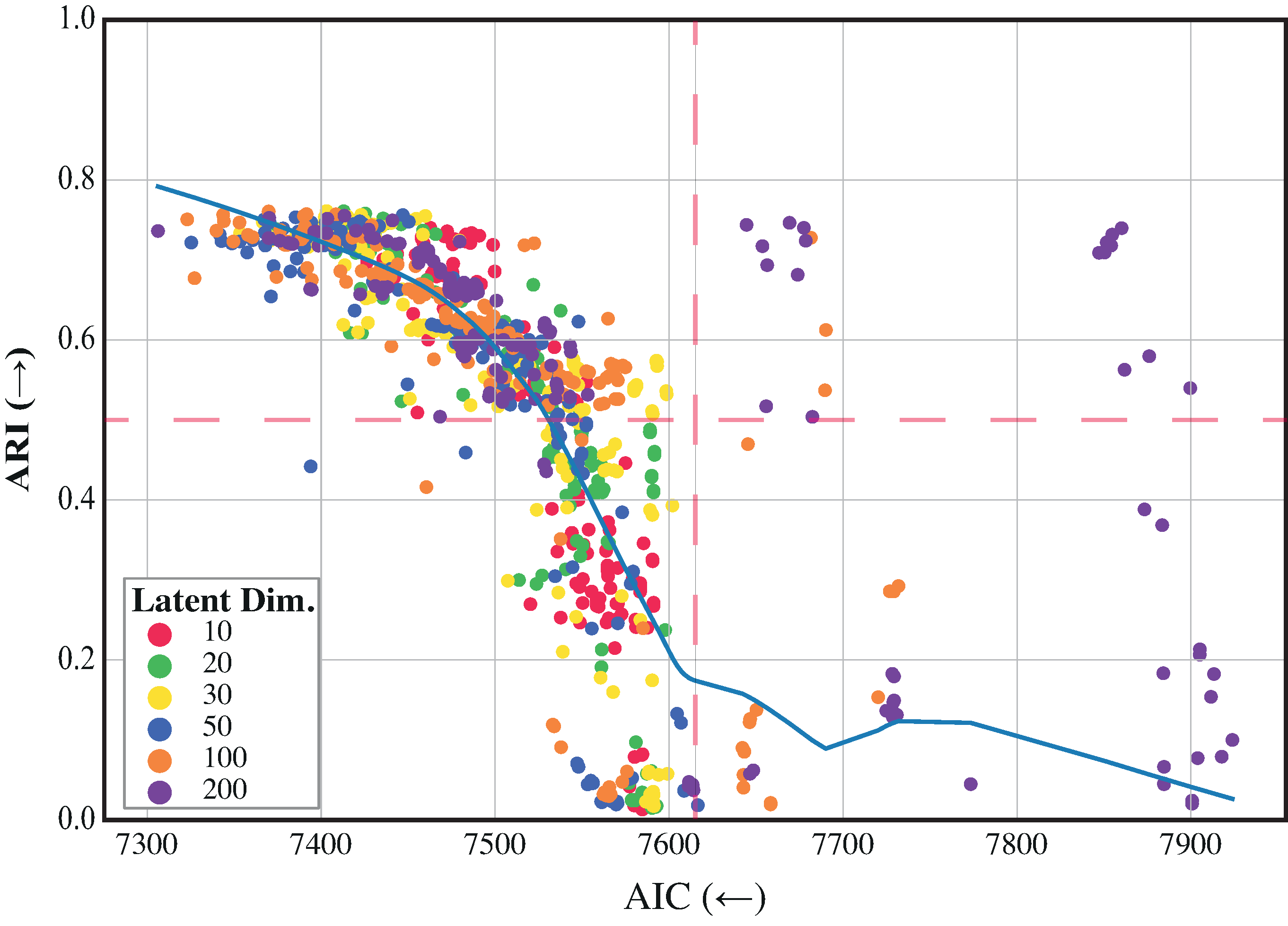

Supplement: S6 Fig — Scatter plot for the clustering performance measured in ARI (y−axis, the higher the better) and survival analysis performance measured in AIC (x−axis, the lower the better). The figure demonstrates the concordance between these two measures, indicating that models with higher ARI tend to have lower AIC. The top left quarter of the plot represents the best performing models across both clustering and survival analysis tasks. Blue line represents the lowess curve fitting for the data. (TIF) [file pone.0292126.s006.tif]

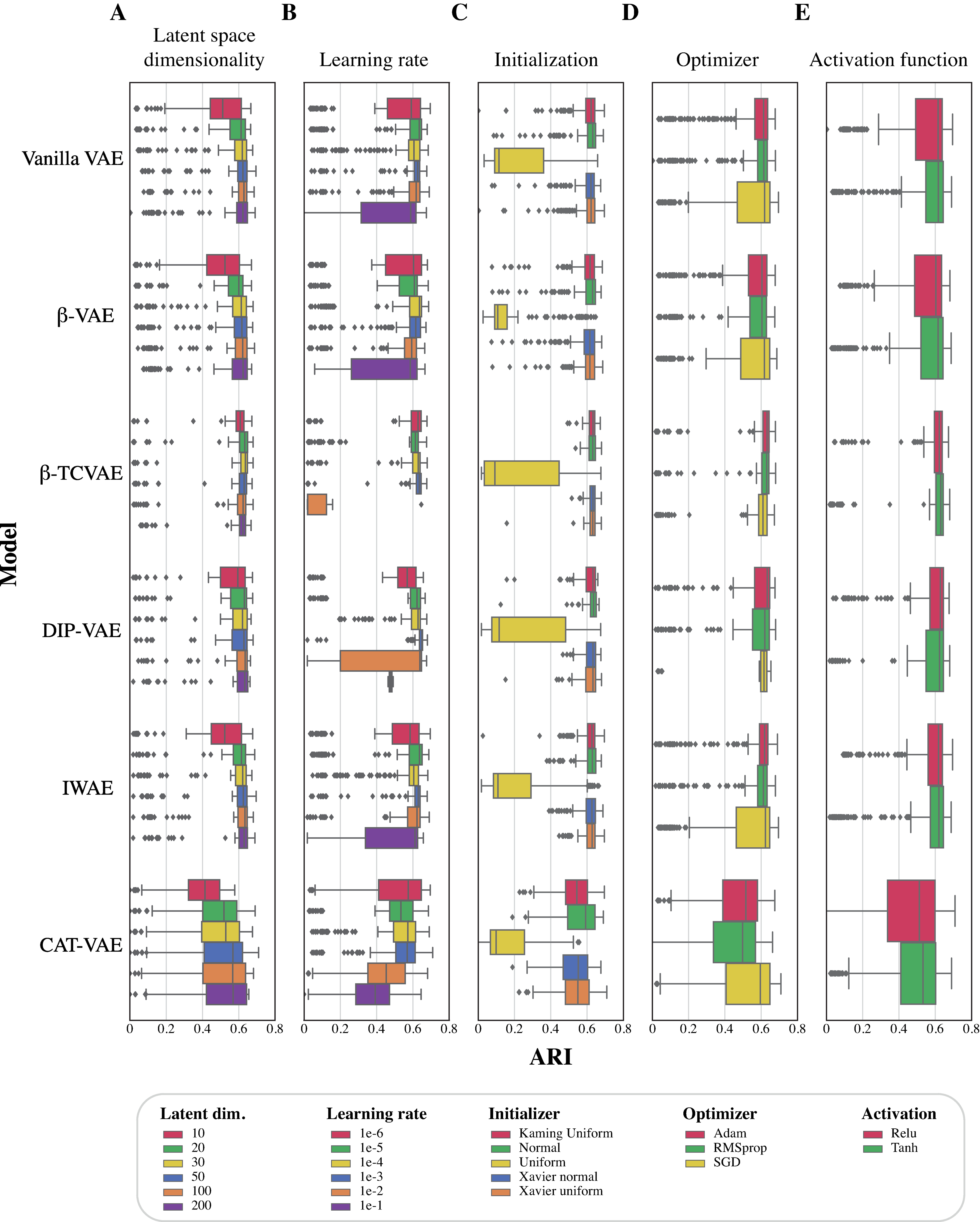

Supplement: S7 Fig — Each boxplot shows the clustering performance (ARI, x−axis) of fixing a hyperparameter while varying all others for each VAE model (y−axis). The five panels show the five different hyperparameters tested: A) Effect of latent dimensions, B) Effect of learning rate, C) Effect of initialization method, D) Effect of optimizer selection, E) Effect of activation layer. The figure shows analogous effect to that found on TCGA dataset. (TIF) [file pone.0292126.s007.tif]

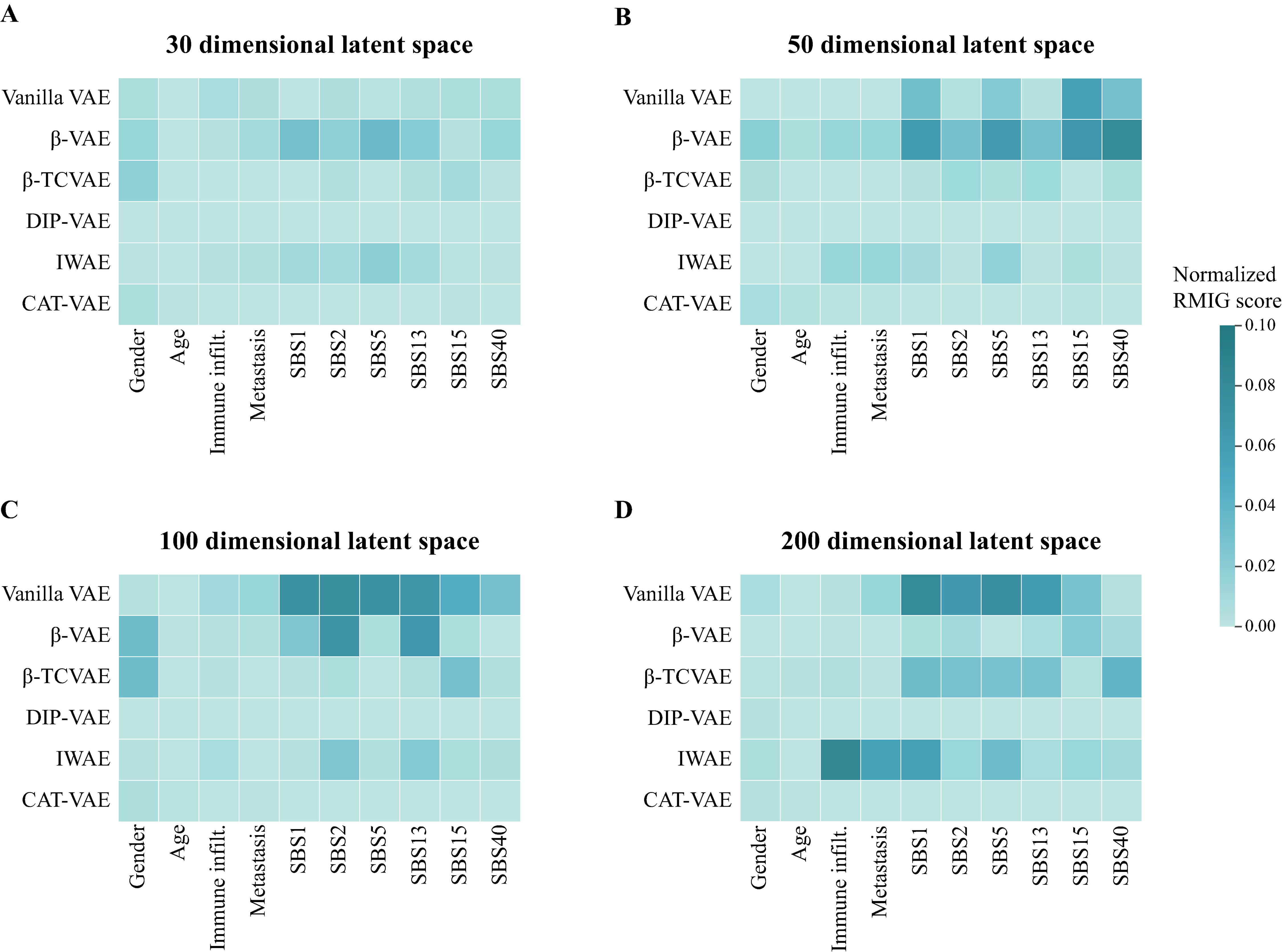

Supplement: S8 Fig — A) Using 30 dimensional latent space. B) Using 50 dimensional latent space. C) Using 100 dimensional latent space. D) Using 200 dimensional latent space. (TIF) [file pone.0292126.s008.tif]
