# Supplementary material for: Benchmarking variational AutoEncoders on cancer transcriptomics data
Source: PLoS One. 2023 Oct 5;18(10):e0292126. doi: 10.1371/journal.pone.0292126 (PMC10553230; doi:10.1371/journal.pone.0292126)
Supplement: S1 Table — A listing of the hyperparameters that were held constant throughout the study. The values were set according to the implementation of https://github.com/AntixK/PyTorch-VAE. (PDF) [file pone.0292126.s009.pdf]

S1 Table. VAE models hyperparameters A listing of the hyperparameters that were held constant throughout the study. The values were set according to the implementation of `https://github.com/AntixK/PyTorch-VAE`.

**Model**	**Parameter**	**Value**
Common parameters	Batch size	64
	Scheduler gamma	0.95
	Weight decay	0.0
	Maximum epochs	1000
β-VAE	β	6
β-TCVAE	β	6
	α	1
	γ	1
DIP-VAE	λ_d	0.05
	λ_{od}	0.1
IWAE	K	5
