# Supplementary material for: Benchmarking variational AutoEncoders on cancer transcriptomics data
Source: PLoS One. 2023 Oct 5;18(10):e0292126. doi: 10.1371/journal.pone.0292126 (PMC10553230; doi:10.1371/journal.pone.0292126)
Supplement: S2 Table — The absolute rounded Spearman correlation between all the different configurations tested for each model and both ARI and AIC values achieved by this model in the downstream task. (PDF) [file pone.0292126.s010.pdf]

S2 Table. Spearman correlation between different models validation loss and ARI, AIC The absolute rounded Spearman correlation between all the different configurations tested for each model and both ARI and AIC values achieved by this model in the downstream task.

**Model**	**ARI** ρ	**AIC** ρ
Vanilla VAE	0.53	0.48
β-VAE	0.49	0.42
β-TCVAE	0.48	0.32
DIP-VAE	0.88	0.8
IWAE	0.57	0.52
CAT-VAE	0.35	0.22
